# Mechanism Governing Human Kappa-Opioid Receptor Expression under Desferrioxamine-Induced Hypoxic Mimic Condition in Neuronal NMB Cells

**DOI:** 10.3390/ijms18010211

**Published:** 2017-01-20

**Authors:** Jennifer Babcock, Alberto Herrera, George Coricor, Christopher Karch, Alexander H. Liu, Aida Rivera-Gines, Jane L. Ko

**Affiliations:** Department of Biological Sciences, Seton Hall University, South Orange, NJ 07079, USA; jennifer.candelora@student.shu.edu (J.B.); alberto.herrera@student.shu.edu (A.H.); gc0506@gmail.com (G.C.); chris.karch@LIVE.COM (C.K.); alcolumbia@gmail.com (A.H.L.); aida139@gmail.com (A.R.-G.)

**Keywords:** desferrioxamine, hypoxia, hypoxia inducible factor-1α, human kappa-opioid receptor, HIF response elements, human NMB neuronal cells

## Abstract

Cellular adaptation to hypoxia is a protective mechanism for neurons and relevant to cancer. Treatment with desferrioxamine (DFO) to induce hypoxia reduced the viability of human neuronal NMB cells. Surviving/attached cells exhibited profound increases of expression of the human kappa-opioid receptor (hKOR) and hypoxia inducible factor-1α (HIF-1α). The functional relationship between hKOR and HIF-1α was investigated using RT-PCR, Western blot, luciferase reporter, mutagenesis, siRNA and receptor-ligand binding assays. In surviving neurons, DFO increased HIF-1α expression and its amount in the nucleus. DFO also dramatically increased hKOR expression. Two (designated as HIFC and D) out of four potential HIF response elements of the *hKOR* gene (HIFA–D) synergistically mediated the DFO response. Mutation of both elements completely abolished the DFO-induced effect. The CD11 plasmid (containing HIFC and D with an 11 bp spacing) produced greater augmentation than that of the CD17 plasmid (HIFC and D with a 17 bp-spacing), suggesting that a proper topological interaction of these elements synergistically enhanced the promoter activity. HIF-1α siRNA knocked down the increase of endogenous HIF-1α messages and diminished the DFO-induced increase of hKOR expression. Increased hKOR expression resulted in the up-regulation of hKOR protein. In conclusion, the adaptation of neuronal hKOR under hypoxia was governed by HIF-1, revealing a new mechanism of hKOR regulation.

## 1. Introduction

The mammalian circulatory system has evolved to efficiently propel oxygen-rich blood. However, traumatic events and diseases, such as myocardial infarction, stroke, diabetes and cancer, can reduce oxygen availability by decreasing blood flow or increasing the demand for oxygen. Among various types of cells, neuronal cells are most susceptible to low levels of oxygen. Hypoxia (low oxygen condition) can decrease ATP production. Thus, neuronal cells cannot efficiently maintain membrane potential (which requires ATP), a condition that can trigger membrane depolarization, calcium influx, and even cell death [1]. Nonetheless, some neurons still survive by developing adaptive responses to overcome the hypoxic challenge [2,3,4,5,6]. Elucidating these cellular adaptation mechanisms to hypoxia is important for understanding neuronal protection as well as for cancer, as hypoxia has effects on cancer progression [5].

Hypoxia is known to increase the expression of hypoxia inducible factor-1 (HIF-1), a transcription factor, which can up- and down-regulate expressions of various genes in response to insult [7,8,9,10]. HIF-1 consists of two subunits; the constitutively expressed HIF-1β and HIF-1α, which is sensitive to oxygen availability. Post-translational modification by hydroxylation of HIF-1α occurs under normal oxygen tension. Prolyl-hydroxylase domain proteins (PHD) 1–3 are responsible for hydroxylating HIF-1α, which is required for the interaction of HIF-1α with the Von Hippel Lindau tumor suppressor protein (VHL) [11,12]. Once in contact with VHL, the recognition component of the E3 ubiquitin ligase complex, HIF-1α is targeted for degradation. Under hypoxia, HIF-1α is not degraded. It accumulates and translocates to the nucleus to form a complex with HIF-1β and alters the transcription of target by binding to the hypoxia response element. Therefore, the increase of HIF-1α mRNA expression can be used as a molecular indicator of a hypoxic environment [13].

To investigate adaptive responses in human neuronal cells under hypoxic condition, a human neuronal cell line (NMB) and a hypoxic mimetic compound, desferrioxamine (DFO), were used to create a hypoxic mimic neuronal cell model system [6]. DFO, one of the commonly used hypoxia mimic compounds, has been shown to simulate the hypoxia condition by increasing the expression of HIF-1α [14,15,16]. Previously, we reported a decrease of cell viability using this DFO-induced hypoxia model system. However, there were still surviving/attached neurons, which displayed no morphological difference from that of non-treated control cells via annexin-V-fluorescein and propidium iodide staining. An increase of HIF-1α expression was also detected in surviving cells [6].

Furthermore, patients suffering from hypoxia can experience pain sensation, which may be managed with opioids [17]. There are three main types of opioid receptors; mu (MOR), delta (DOR), and kappa (KOR), all of which are mainly expressed in the nervous system [18] and are seven transmembrane G-protein coupled receptors (GPCRs) with a variety of functionalities dependent on stimuli, environment, and cell type [19]. Using this DFO-induced hypoxic cell model, we previously reported that there was no significant change in DOR expression but a decrease of MOR expression in surviving neurons. However, no HIF response elements were found in the MOR core promoter region, suggesting that HIF-1 is not directly controlling MOR expression [6]. In this study, we further reported the alteration of KOR expression and deciphered its underlying mechanism using DFO-induced hypoxic mimic condition with human neuronal NMB cells.

## 2. Results

### 2.1. Increase of Neuronal hKOR Gene Expression under DFO Challenge

Human NMB neuronal cells [20], endogenously expressing mu-opioid receptors (hMOR), the delta-opioid receptor (hDOR), and the kappa-opioid receptor (hKOR), were treated with desferrioxamine (DFO) to create a DFO-induced hypoxic mimic condition. Previously, using this cell model system we reported that 24 h DFO treatment resulted in a certain degree of cell death (about 30%), while surviving/attached neuronal cells developed adaptive responses, such as an increase of HIF expression and a decrease of hMOR expression with no significant change found in the hDOR expression [6]. Here, we reported that the percentage of cell survival rate was 66% ± 5.5% (The number of control cells was arbitrarily defined as 100%) using NMB cells treated with DFO (300 μM) for 24 h. The surviving/attached cells also showed no morphology different from the non-treated control cells used via annexin-V-fluorescein and propidium iodide staining, in agreement with our previous report [6].

**Figure 1:**
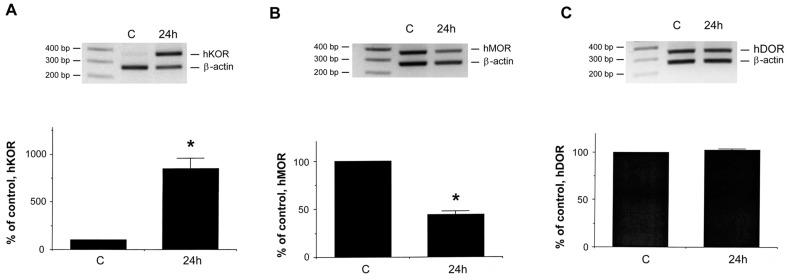
Effect of desferrioxamine (DFO) on opioid receptor gene expression in NMB cells. Cells were treated without (control; C) or with 300 μM DFO for 24 h. Dead cells were removed. The surviving/attached cells were harvested. RNAs from cells treated without (C) or with DFO for 24 h were extracted. Semi-quantitative RT-PCR was carried out using a pair of human KOR ((**A**) hKOR); MOR ((**B**) hMOR); or DOR ((**C**) hDOR)-specific primers. Human β-actin specific primers, as the internal standard, were also used in PCR reaction (added at the cycle 19) for normalization. The normalized message of control (non-DFO treated sample) was designated as 100%. Data is presented as mean ± S.E.M. Experiments were repeated eight times with similar results. “*” indicates *p* < 0.01. (Student *t*-test).

In this study, we further investigated the effect of DFO on hKOR expression using this cell model system. Semi-quantitative RT-PCR was performed using a specific pair of primers for hKOR and β-actin, which was used as an internal standard for normalization. For comparison, the levels of hMOR and hDOR were also examined. As shown in Figure 1A, treatment with DFO (300 μM) for 24 h increased the hKOR expression significantly. Simultaneously, a decrease of hMOR expression (Figure 1B) and no significant change of hDOR expression (Figure 1C) were also observed, confirming our previous reports [6]. Taken together, these results showed that the DFO-induced hypoxic mimic condition differentially influenced opioid receptor expression, with *hKOR* gene expression significantly increasing in surviving/attached neurons.

### 2.2. Increase of HIF-1α Amount in the Nucleus of Survival Cells under DFO Challenge

**Figure 2:**
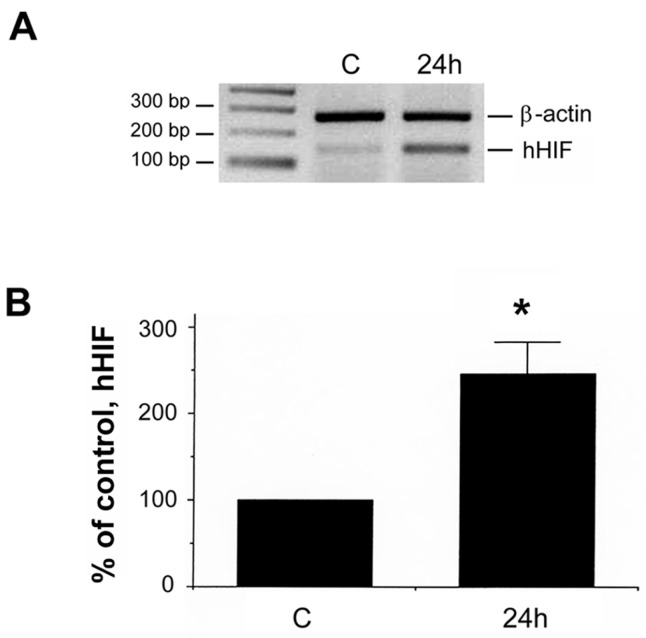

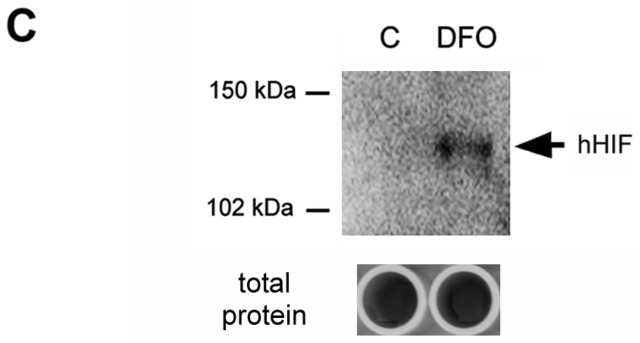
Validation of DFO effect on HIF-1α in NMB cells. (**A**) Cells were treated without or with DFO for 24 h. Dead cells were removed. The surviving/attached cells were harvested. RNAs were extracted from cells treated without (control; C) or with DFO for 24 h. Semi-quantitative RT-PCR was performed using a pair of human HIF-1α (hHIF) specific primers. A pair of human β-actin specific primers, as an internal standard, was also included in PCR reaction (added at the cycle 19) for normalization use; (**B**) the normalized hHIF-1α message from the non-DFO treated control was designated as 100%. Data is presented as mean ± S.E.M. Experiments were repeated eight times. “*” indicates *p* < 0.01. (Student *t*-test); (**C**) Western blot analysis was performed using an anti-hHIF-1α antibody with the same amount of nuclear extracts from control (C) or DFO treated cells (DFO). The amount of total proteins for each sample was shown in the panel below using colormetric Lowry assay. The hHIF-1α signal (indicated by an arrow) was detected only in DFO treated cells, demonstrating its nuclear translocation.

We also determined the effect of DFO on the expression of hypoxic-inducible factor 1α (HIF-1α) transcription factor. As shown in Figure 2A,B, using semi-quantitative RT-PCR, the increase of HIF-1α expression (hHIF) was detected under DFO challenge, which demonstrated that surviving cells developed the hypoxic condition, in agreement with our previous report [6]. To further verify if the transcription factor, HIF-1α, indeed translocated into the nucleus upon DFO treatment, Western blot analysis with an anti-HIF-1α antibody was performed using nuclear extracts (40 μg of total proteins) from DFO treated or non-treated control cells. As shown in Figure 2C, the HIF-1α band (hHIF; indicated by an arrow) was detected only in DFO treated cells (DFO) but not in control cells (C), demonstrating the nuclear translocation of the HIF-1α transcription factor in surviving NMB cells upon DFO challenge. 

### 2.3. Effect of HIF Response Elements of hKOR Gene on the Promoter Activity upon DFO Treatment

Since HIF-1α is a transcription factor, which can up- or down-regulate the gene expression, we therefore examined about 10 Kb of DNA sequences located at the 5′-upstream of the start codon (ATG) of the *hKOR* gene to identify potential HIF response elements (NCBI database). Four potential HIF response elements were identified (The consensus sequences are underlined). These were designated as HIF A (5′-GGGATTACAGGCGTGAGCCATCACAC-3′; about 9.5 Kb upstream from the start codon of the *hKOR* gene), HIF B (5′-CCACACCACCACGTGTCAGGCTCTCA-3′; about 3.7 Kb upstream), HIF C (5′-GTGAGGAGAACGTGATGGCTGCAGGGA3′; about 1 Kb upstream), and HIF D (5′-GTAGTGGGAGACGTGCGCTGAGAGGC-3′; about 550 bp upstream), respectively. Each potential HIF response element was synthesized and cloned into the pGL3-promoter plasmid (P), containing the luciferase reporter gene and SV40 promoter. A positive control (HIF-NOS plasmid) was also generated, containing the HIF response element of the NO synthetase gene [21]. Resulting plasmids were subjected to DNA sequencing to verify correct sequences. 

**Figure 3:**
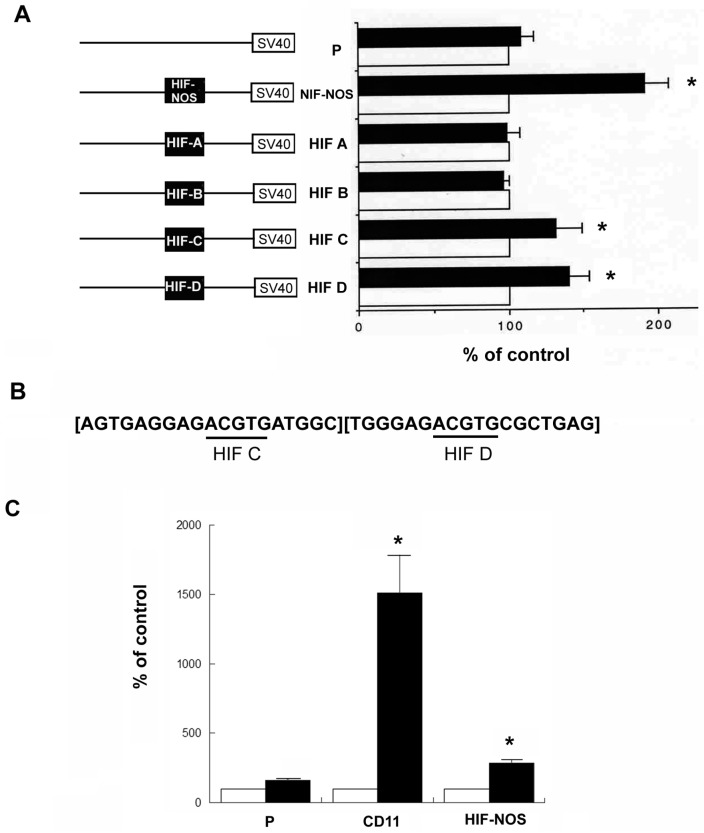
Identification of functional hypoxia inducible factor (HIF) response elements located at 5′-upstream of the start codon of the *hKOR* gene under DFO treatment. (**A**) Based on the consensus sequence comparison, four potential HIF response elements located at the 5′-upstream of the start codon of the *hKOR* gene were identified. These HIF response elements were individually cloned into the pGL3-promoter vector (P), containing SV40 promoter and the luciferase reporter gene. Resulting plasmids were designated as HIF A–D. A positive control, HIF-NOS plasmid containing the HIF response element of the NO synthetase gene [21], was included. All plasmids were subjected to DNA sequencing to validate correct sequences. Transient transfection was performed using NMB cells. The pCH110 plasmid, containing the β-galactosidase, was also transfected simultaneously, and its activity was used as the internal standard for normalization purpose. Twenty-four hours after transfection, cells were treated without or with DFO for an additional 24 h. Cells were then harvested and luciferase assays were performed. The promoter activity of each construct with no DFO treatment (as the control) was arbitrarily defined as 100% (white bars). The activity of each construct under DFO treatment (black bars) was presented as % of control activity. Histograms represent mean values of activation. Error bars indicate S.E.M. Experiments were repeated at least ten times. “*” indicates *p* < 0.01 (student *t*-test); (**B**) sequences of the combined HIF C (left side) and D (right side) elements are depicted. The HIF consensus sequences are underlined. There is an 11 bp spacing between the underlined HIF C and D elements; (**C**) NMB cells were transfected with the CD11 plasmid (containing both HIF C and D elements), the vector (pGL3-promoter), and the positive control (HIF-NOS), respectively. The pCH110 plasmid with β-galactosidase gene was also included as an internal standard for normalization. Twenty-four hours after transfection, cells were treated without (control; white bars) or with DFO for an additional 24 h (black bars). Cells were harvested and luciferase assays were performed. The promoter activity of each construct under DFO treatment (black bars) was expressed as % activity of the non-DFO treated control, arbitrarily defined as 100% (white bars). Histograms represent mean values of activation. Error bars indicate S.E.M. Experiments were repeated at least six times. “*” indicates *p* < 0.01 (Student *t*-test).

NMB cells were then transfected with these plasmids, respectively. The pCH110 plasmid, containing the β-galactosidase gene, was also co-transfected simultaneously for normalization purpose. Transfected cells were then treated with DFO for 24 h (black bars). The non-treated cells were used as the control (open bars; arbitrarily defined as 100%). As shown in Figure 3A, upon DFO treatment (black bar), a significant increase of the normalized activity was observed using HIF-NOS plasmid (the positive control) as compared to the non-treated HIF-NOS control (open bar). Similar results were observed using HIF C and HIF D plasmids, but not using the HIF A, HIF B plasmid or vector itself (P). These results demonstrated that two HIF response elements (HIF C and HIF D), located close to the start codon of the *hKOR* gene, can enhance the promoter activity. 

To determine if the combination of HIF C and HIF D can produce an additive or synergistic effect on the promoter activity, a fragment containing both HIF C and D consensus sequence (CD) with a 11-bp spacing (Figure 3B; HIF consensus sequences were underlined) was cloned into the vector (pGL3-promoter). This resulting plasmid (CD11) was authenticated via DNA sequencing. NMB cells transfected with the plasmid were treated without (controls; white bars) or with DFO (black bars). Simultaneously, the vector (P) and HIF-NOS plasmids were also tested. As shown in Figure 3C, the activity of the CD11 plasmid was dramatically increased as compared to that of the vector (P) or HIF-NOS plasmid upon DFO treatment. This result demonstrated that the combination of HIF C and D elements enhanced the promoter activity synergistically. 

**Figure 4:**
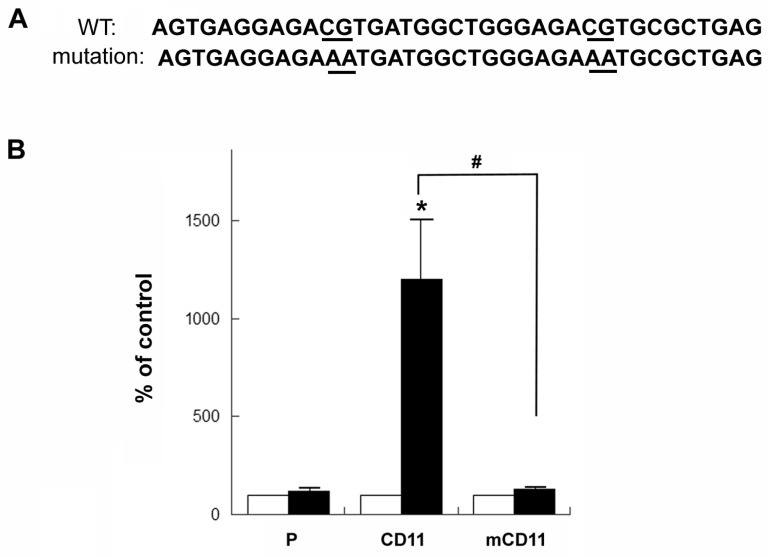

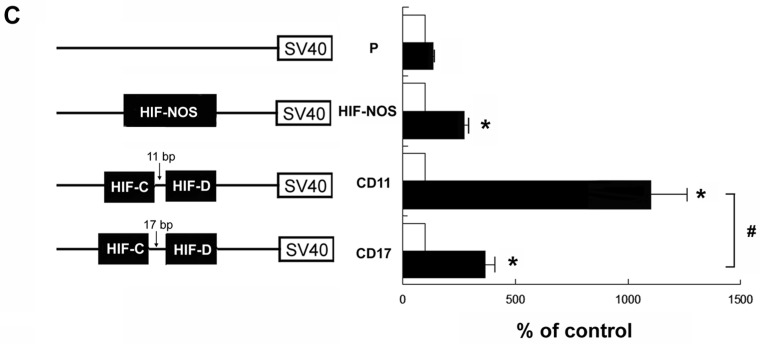
Effects of mutation and spacing of HIF C and D response elements of the *hKOR* gene on the luciferase reporter activity under DFO treatment. (**A**,**B**) Validation of the functional role of HIF C and D elements upon DFO challenge using the mutation analysis. The HIF consensus sequences were mutated to AA (depicted in (**A**) by underlines). The mutated fragment was cloned into the vector (pGL3-promoter). The resulting mutant plasmid (mCD11) was validated by DNA sequencing. NMB cells were transfected with the mCD11, CD11, and the pGL3-promoter vector (P), respectively. The pCH110 plasmid with the β-galactosidase was co-transfected and used as an internal standard. Transfected cells were treated without (control; white bars) or with DFO for 24 h (black bars). Cells were harvested and subjected to luciferase assay. The promoter activity of each construct under DFO treatment (black bars) was expressed as a percentage of non-DFO treated control activity, arbitrarily defined as 100% (white bars). Histograms represent mean values of activation. Error bars indicate S.E.M. Experiments were repeated at least ten times. “*” indicates *p* < 0.01 (student *t*-test). “#” indicates a significant difference (*p* < 0.01; student *t*-test) between the CD11 and mCD11 activities; (**C**) the effect of topological arrangement of HIF C and D elements on the promoter activity under DFO treatment. The HIF C and D elements with a 17 bp spacing were cloned into the vector (pGL3-promoter). The resulting plasmid (CD17) was validated by DNA sequencing. NMB cells were transfected with the CD17, CD11, HIF-NOS, and the pGL3-promoter vector (P), respectively. The pCH110 plasmid as an internal standard was also co-transfected. Transfected cells were treated without (control; white bars) or with DFO for 24 h (black bars). Cells were harvested and luciferase assays were performed. The promoter activity of each construct under DFO treatment (black bars) was expressed as % of non-DFO treated control activity of individual plasmid (white bars), arbitrarily defined as 100%. Histograms represent mean values of activation. Error bars indicate S.E.M. Experiments were repeated at least six times. “*” indicates *p* < 0.01 (student *t*-test). “#” indicates a significant difference (*p* < 0.01; Student *t*-test) in reporter activities between the CD11 and CD17 plasmids.

To further validate the functional role of HIF C and D elements, two-point mutation was introduced (Figure 4A; AA mutation was introduced to replace CG). The resulting plasmid (mCD11) was confirmed using DNA sequencing. The mCD11 and CD11 plasmids were then tested using transient transfection in NMB cells. As shown in Figure 4B, the mCD11 plasmid displayed no significant difference in the promoter activity upon DFO treatment (black bar) vs. non-treated group (control; open bar), while the CD11 plasmid showed an increase of promoter activity upon DFO treatment. These results showed that the mutation of HIF C and D elements abolished their abilities to enhance the promoter activity upon DFO challenge.

To examine if a different spacing between HIF C and D elements can influence their synergism, a 17 bp spacing between HIF C and D element was then constructed. The sequences are 5′-AGTGAGGAGACGTGATGGCTGGTAGTGGGAGACGTGCGCTGAGAG-3′ (HIF consensus sequences are underlined). The resulting plasmid (CD17) was confirmed by DNA sequencing. The CD17 plasmid was tested using transient transfection with NMB cells, which were then challenged with DFO. The vector (P), CD11 and HIF-NOS plasmids were also tested. As shown in Figure 4C, upon DFO challenge, the CD17 plasmid displayed a much lower promoter activity as compared to that of the CD11 plasmid, though its activity was similar to that of HIF-NOS plasmid. 

In summary, these results suggested that two HIF elements (C and D), closed to the start codon of the *hKOR* gene, can work synergistically to enhance the promoter activity, and the arrangement between these two elements can shape the efficiency of hKOR promoter activity.

### 2.4. Effect of HIF-1α siRNA on the hKOR Gene Expression under DFO Challenge

The above results suggested that HIF response elements participated in the regulation of *hKOR* gene expression under DFO treatment. To determine if HIF-1α indeed regulated the increase of hKOR expression in NMB cells upon DFO treatment (Figure 1A), cells were transfected with HIF-1α siRNA (10 or 20 nM). After 24 h transfection, cells were treated without or with DFO for an additional 24 h. RNAs, extracted from cells, were subjected to semi-quantitative RT-PCR using primers specific for hHIF-1α and β-actin, which was used as an internal standard for normalization. 

**Figure 5:**
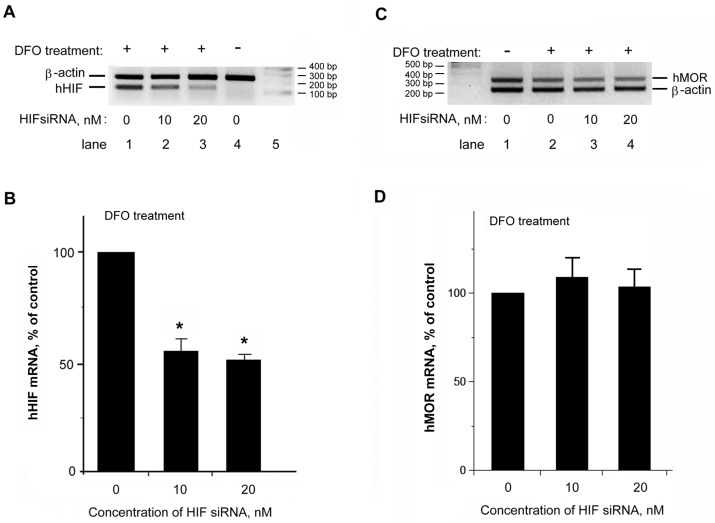
Examined effects of hHIF-1α siRNA on the levels of hHIF-1α and hMOR mRNAs. (**A**) NMB cells were transfected with hHIF-1α siRNA (lane 2, 10 nM; lane 3, 20 nM) or without hHIF-1α siRNA (indicated as 0 nM; lanes 1 and 4). Twenty-four hours after transfection, cells were treated without (lane 4, −) or with DFO for 24 h (lanes 1–3, +). RNAs were extracted from transfected cells. Semi-quantitative RT-PCR was carried out by using a pair of human hHIF-1α (hHIF) primers. The β-actin primers, as the internal standard, were also added to PCR reaction for normalization use. PCR products were separated by gel electrophoresis. Lane 5: DNA markers; (**B**) quantitation of human hHIF-1α mRNA levels is shown, with the normalized hHIF-1α mRNA level from the DFO treated sample without siRNA as 100%. The hHIF-1α siRNA significantly knocked down the endogenous hHIF-1α mRNA. Histograms represent mean ± S.E.M. Experiments were repeated eight times. Asterisk ‘‘*” indicates *p* < 0.01 (*t*-test); (**C**) NMB cells were transfected with hHIF-1α siRNA (lane 3, 10 nM; lane 4, 20 nM) or without hHIF-1α siRNA (0 nM in lanes 1 and 2). Twenty-four hours after transfection, cells were treated without (lane 1, −) or with DFO for 24 h (lanes 2–4, +). Semi-quantitative RT-PCR was carried out by using a pair of hMOR primers, along with a pair of β-actin primers as the internal standard for normalization use. PCR products were separated by gel electrophoresis. Lane 1: DNA markers; (**D**) quantitation of hMOR mRNA levels is shown, with the normalized hMOR mRNA level from the DFO treated sample without siRNA as 100%. The hHIF-1α siRNA treatment did not alter the endogenous level of hMOR mRNA. Histograms represent mean ± S.E.M. Experiments were repeated eight times.

As shown in Figure 5A, a significant DFO-induced increase of HIF-1α mRNA level (lane 1; labeled as hHIF) was observed as compared to that of the non-treated control (lane 4), while β-actin levels (an internal standard for normalization) were similar in both samples. These data confirmed that DFO treatment increased the HIF-1α mRNA level in NMB cells. Furthermore, both concentrations (10 and 20 nM) of HIF-1α siRNA (lanes 2 and 3) significantly reduced the increase of HIF-1α mRNA level upon DFO challenge. The normalized HIF-1α amounts were quantitated and shown in Figure 5B. The level of HIF under DFO with no HIF-1α siRNA was arbitrarily defined as 100%. Taken together, results demonstrated that HIF-1α siRNA effectively knocked down the increase of the endogenous HIF mRNA level upon DFO challenge.

To verify if the decrease of the endogenous HIF-1α mRNA level by HIF siRNA also affected the DFO-induced increase of hKOR mRNA, the semi-quantitative RT-PCR was performed using the same RNA preparations with hKOR-specific and β-actin as the internal standard. For comparison, the siRNA effect on the DFO-induced decrease of hMOR mRNA was also tested. Results (Figure 5C) showed that DFO induced a decrease of hMOR mRNA (lane 2) in comparison to that of the non-treated sample (lane 1), agreeing with our previous results [6]. However, the DFO-induced decrease of hMOR mRNA was not influenced by knocking down the HIF-1α mRNA in either 10 or 20 nM HIF-1α siRNA treated samples (lanes 3 and 4). The quantitative data was shown in Figure 5D, with the normalized hMOR mRNA under DFO with no HIF siRNA as 100%. Results suggested that the decrease of hMOR mRNA under DFO treatment was not mediated by HIF, in agreement with our previous observation [6]. 

**Figure 6:**
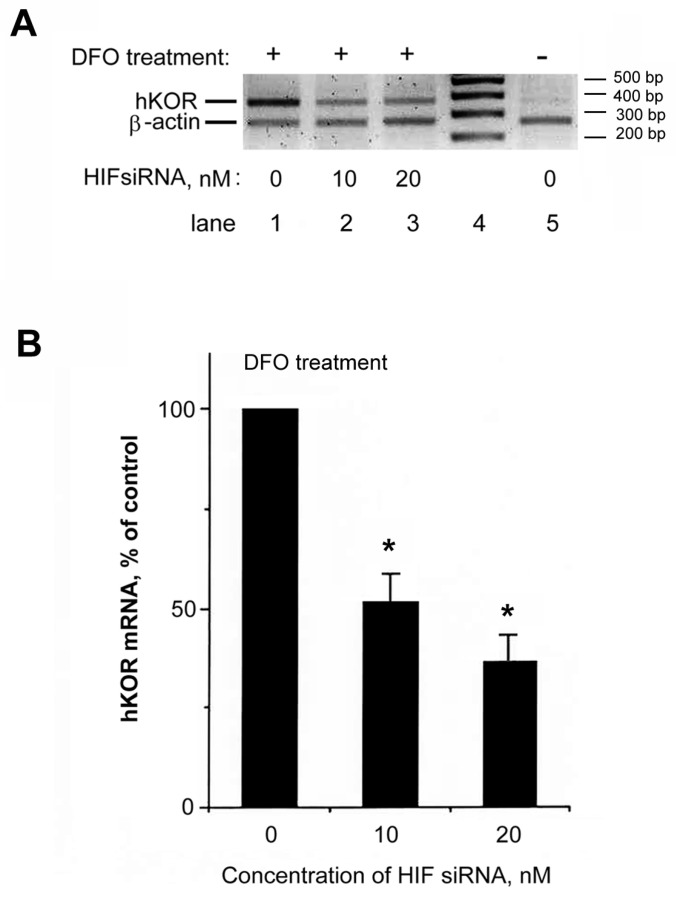
Examination of the effect of hHIF-1α siRNA on hKOR expression. (**A**) The hHIF-1α knockdown attenuated DFO-induced enhancement of hKOR expression. NMB cells were transfected with hHIF-1α siRNA (lane 2, 10 nM; lane 3, 20 nM) or without hHIF-1α siRNA (0 nM; lanes 1 and 5). Twenty-four hours after transfection, cells were treated without (lane 5, −) or with DFO for 24 h (lanes 1–3, +). Semi-quantitative RT-PCR was performed using a pair of primers specific to hKOR with β-actin specific primers as the internal standard for normalization. PCR products were analyzed using gel electrophoresis. Lane 4: DNA markers (each maker was labeled on the right-hand side); (**B**) quantitation of hKOR mRNA levels is shown, with the normalized hKOR mRNA level from the DFO treated sample without siRNA as 100%. The hHIF-1α siRNA treatment significantly diminished the DFO-induced increase of endogenous hKOR mRNA. Histograms represent mean ± S.E.M. Experiments were repeated eight times. Asterisk ‘‘*” indicates *p* < 0.01 (*t*-test).

Importantly, HIF-1α siRNA not only knocked down the HIF-1α mRNA expression, but also diminished the increase of hKOR expression upon DFO treatment (Figure 6A, lane 2 with 10 nM siRNA and lane 3 with 20 nM siRNA) as compared to that of the DFO treated sample with no HIF-1α siRNA (lane 1). These results also validated that DFO treatment increased hKOR expression, as compared to the DFO treated group (lane 1) vs the non-DFO treated group (lane 5). The quantitative data is shown in Figure 6B, with the normalized hKOR message under DFO with no HIF siRNA as 100%. In conclusion, these results demonstrated that upon DFO challenge, the increase of hKOR expression was mediated directly by the HIF-1α transcription factor in NMB cells. 

### 2.5. Increase of hKOR Ligand Binding in NMB Cells upon DFO Challenge

To determine if the increase of hKOR mRNA level upon DFO treatment also resulted in an increase of the hKOR receptor protein level, the receptor-ligand binding assay was performed. The radioisotope ligand [^3^H]-diprenorphine, along with 10 μM of DAMGO and DPDPE each to block the binding of MOR and DOR, respectively, were used. After 24 h DFO treatment, an increase of hKOR binding was observed but did not reach statistical significance. We therefore extended the DFO treatment up to 48 h, examining mRNA levels of hKOR at different time points first, simultaneously testing hMOR and hDOR messages for comparison, using the semi-quantitative RT-PCR. 

**Figure 7:**
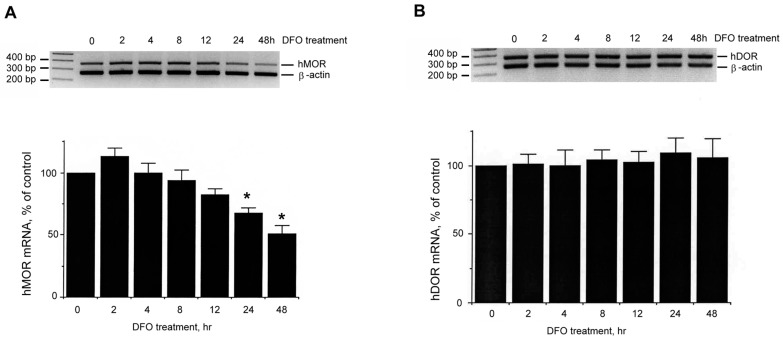
Effect of DFO on *hMOR* and *hDOR* gene expression at various time points. Cells were treated without (control; indicated as “0”) or with DFO for various time points (2 to 48 h). RNAs from surviving/attached cells were extracted. Semi-quantitative RT-PCR was performed using hMOR (**A**); or hDOR (**B**)-specific primers. Human β-actin specific primers were included as an internal control for normalization. The normalized message from the non-DFO treated control was arbitrarily defined as 100%. Quantitative analysis of message levels is presented as mean ± S.E.M. Experiments were repeated at least six times. “*” indicates *p* < 0.01. (Student *t*-test).

As shown in Figure 7A, a significant decrease of hMOR mRNA level (hMOR) was still observed up to 48 h DFO treatment as compared to the non-DFO treated group (control; labeled as 0). No significant change of hDOR mRNA was observed (Figure 7B), while the internal standard, β-actin messages (β-actin), remained at the similar levels. Quantitative data of normalized hMOR and hDOR messages are shown in the bar graphs below, respectively (non-DFO treated control as 100%). 

**Figure 8:**
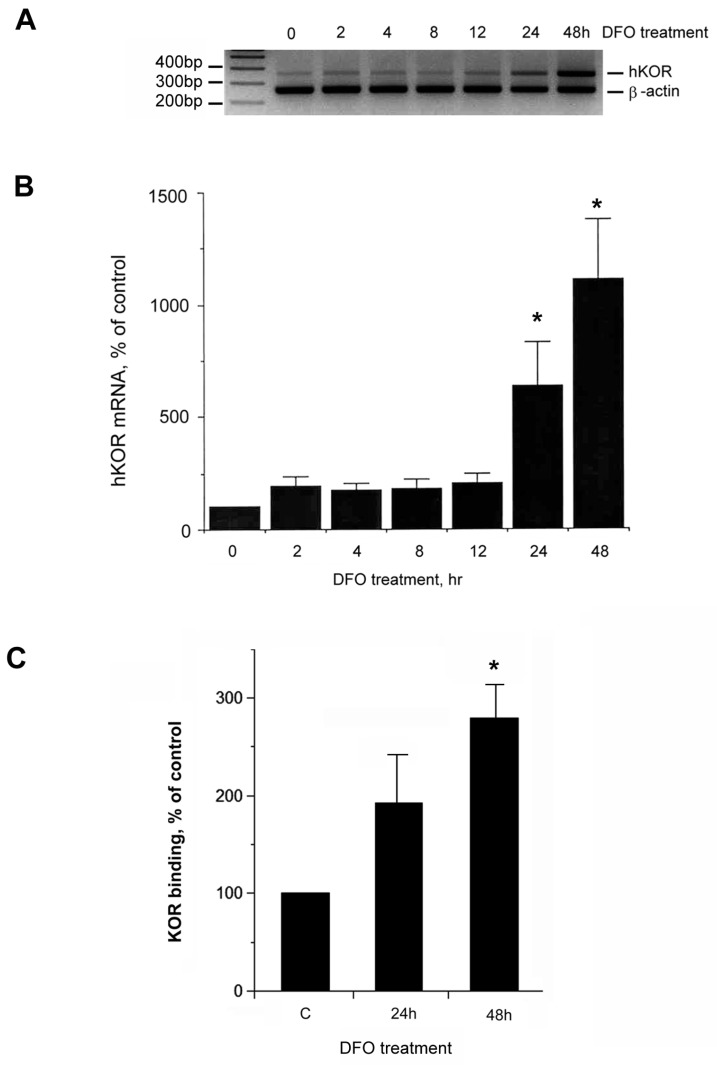
Effect of DFO on hKOR expression at various time points. (**A**) Cells were treated without (control; indicated as “0”) or with DFO for various different time points (2 to 48 h). RNAs from surviving/attached cells were extracted. Semi-quantitative RT-PCR was performed using hKOR-specific primers with human β-actin specific primers as an internal control for normalization; (**B**) the normalized message from the non-DFO treated control (labeled as “0”) was arbitrarily defined as 100%. Quantitative analysis of message levels are presented as mean ± S.E.M. Experiments were repeated at least ten times. “*” indicates *p* < 0.01. (Student *t*-test); (**C**) the level of hKOR proteins was determined by the receptor-ligand binding assay. Two nM of [^3^H]-diprenorphine was used as the labeled ligand, and U50, 488H (1 µM) was included as the competitive ligand. In order to block the bindings to delta and mu opioid receptors, 1 µM of DAMGO and DADLE were also added to all reactions. Membranes from NMB cells treated without DFO (control; C) or with DFO for 24 or 48 h were used for the competitive binding assay. Specific binding was denoted as the difference between samples in the absence and presence of 1 μM U50, 488H. Specific [^3^H]-diprenorphine binding of the control (the non-DFO treated sample) was designated as 100%. The binding was normalized by the same amount of proteins. Histograms of KOR bindings are presented as mean ± S.E.M. Experiments were repeated six times. Asterisk ‘‘*” indicates *p* < 0.01 (*t*-test).

As shown in Figure 8A, a significant increase of the hKOR mRNA level was observed up to 48 h DFO treatment as compared to that of the non-treated control (labeled as 0). Normalized data using β-actin as an internal standard is shown in Figure 8B (control as 100%). 

The KOR receptor binding assay was then performed using cell membranes with the same amount of proteins prepared from NMB cells treated without (control; labeled as C) or with DFO treatment. The [^3^H]-diprenorphine ligand along with 10 μM of DAMGO and DPDPE each to block the binding of MOR and DOR were used. The specific hKOR binding was calculated using the difference between the absence and presence of the KOR ligand, U50, 488H. The specific count from the control sample was arbitrarily defined as 100%. A significant increase of hKOR binding was observed at 48 h DFO treatment, but not at the 24 h time point (Figure 8C). These results demonstrated that an increase of hKOR mRNA resulted in an increase of hKOR protein upon DFO treatment. 

## 3. Discussion

Using DFO-induced hypoxia to the hypoxic mimic condition with human neuronal NMB cells, we report a newly identified cellular adaptive response, a dramatic upregulation of hKOR expression (Figure 1A). DFO treatment increased HIF-1α expression, in agreement with our previous report [6], and consistent with an increase of HIF-1α mRNA expression mediated by direct hypoxia (1% O_2_) [22]. The increase of HIF-1α expression enhanced its nuclear translocation (Figure 2) using this model system. Therefore, a functional relationship between the transcription factor, HIF-1α, and hKOR was elucidated in this study. 

Although four potential HIF response elements (HIF A, B, C and D) located at the 5′-upstream of the start codon of the *hKOR* gene were identified, only two elements (HIF C and D) close to the start codon displayed a significant enhancement of promoter ability upon DFO challenge using the luciferase reporter assay (Figure 3A). These two HIF response elements (C and D) worked in concert to increase the promoter activity synergistically (Figure 3C), and two-point mutation of HIF consensus sequences completely abrogated their enhancement of activity upon DFO treatment (Figure 4B), confirming the enhancer function of HIF C and D response elements of the *hKOR* gene. In addition, the CD11 plasmid, containing HIF C and D elements with an 11 bp spacing, conferred a stronger activity (a synergistic effect) than that of a 17 bp spacing (an additive effect), CD17 plasmid (Figure 4C). The 11 bp spacing corresponds to about one turn of the DNA double helix (approximately 10.5 bp per turn), suggesting that the topological relationship of HIF C and D can shape their transactivation ability. This arrangement of HIF C and D elements is likely to manifest itself in a head to tail or tail to head manner, but not head to head or tail to tail way, in order to synergistically augment the promoter activity. 

We also demonstrated that HIF mediated hKOR expression upon DFO challenge using HIF-1α siRNA. The HIF-1α siRNA knock-down strategy not only reduced the DFO-induced increase of endogenous HIF-1α messages (Figure 5A,B) but also reduced the increase of hKOR expression (Figure 6A,B). Together, these data (Figure 3, Figure 4, Figure 5 and Figure 6) clearly demonstrated that the mechanism underlying the powerful enhancement of hKOR expression in DFO-induced hypoxia is HIF-mediated. Furthermore, using the HIF siRNA strategy, there was no significant effect on the DFO-induced decrease of hMOR expression (Figure 5C,D), demonstrating that HIF does not directly regulate hMOR expression under DFO challenge, in agreement with our previous finding that no HIF response element was found in the hMOR core promoter region [6]. This hMOR change may be modulated via different pathways, such as an epigenetic event [23].

The increase of the hKOR message also resulted in an increase of hKOR protein (Figure 8). Using the receptor-ligand binding assay, an increase was observed at 24 h DFO treatment, though its increase was significant only at 48 h DFO challenge, whereas using RT-PCR, a significant increase of hKOR messages was observed at 24 h treatment already. It would be expected that a change in translation would take a longer time than a change in transcription, though the difference can also reflect different sensitivities of these two methods. 

DFO creates hypoxia by chelating irons [14] and altering the iron status of iron- and O_2_-dependent hydroxylases, from which HIF receives information about the cellular O_2_ level. Based on the literature [24,25], HIF-1 activation by hypoxia occurs at oxygen levels less than 5%. Therefore, DFO treatment likely created an O_2_ level lower than 5%. We employed 300 μM DFO to create the hypoxic mimic condition in this study. However, 100 and 200 μM as well as 300 μM concentrations of DFO also produced significant increases of reporter activities, as shown using the CD11 plasmid, containing HIF C and D elements of the *hKOR* gene, as compared to those of the non-treated control group or the blank vector groups (Figure S1). No statistical differences were found among the effects of these three treatments. 

DFO can also be used to treat diseases, such as hemochromatosis by reducing serum ferritin levels as well as hepatic iron levels, and metal ion overload diseases involving aluminum and chromium, and may be a potential treatment for neurodegenerative diseases [26,27,28,29]. Therefore, its iron chelating ability can produce additional effects, such as anti-proliferation and redox [26,27,28,29], which may affect gene regulation. In addition, the responses to different hypoxic mimic compounds can vary in different cell types. There are other hypoxic mimetic compounds, such as DMOG and cobalt, which are commonly used to increase HIF-1 expression and to mimic the hypoxic condition and which can produce additional and some different effects [30,31]. Therefore, we also examined NMB cells using the luciferase reporter assay with CD11 plasmid and cobalt treatment; an increase of reporter activity was also detected in preliminary results (Figure S2). 

In this study we mainly focused on the relationship between HIF-1 and hKOR expression. However, we also tested HIF-2α messages [32] using HIF-1α siRNA treated samples, reported in the Results section. HIF-1α siRNA treatment significantly reduced the level of HIF-1α mRNA, but not of HIF-2α mRNA (Figure S3). Therefore, with no change of the HIF-2α mRNA level, it showed that HIF-2α is not related to the reduction of DFO-induced hKOR mRNA increase; however, the reduction of HIF-1α mRNA is correlated with the reduction of DFO-induced hKOR mRNA increase. Collectively, these results demonstrated that HIF-1 regulates hKOR expression. However, different genes may be regulated by different HIF factors [32,33]. Whether HIF-2 can or cannot regulate hKOR expression (as siRNA HIF-1α only knocked down about a half of endogenous HIF-1a mRNA level) or any other HIF-2 target genes in this hypoxic cell model, will need to be investigated in the future.

KOR is known to be involved in addiction/rewarding, pain modulation and behavioral stress [34,35,36]. Other functional roles of KOR have also been reported. For example, KOR was reported to protect cardiomyocytes from ischemic injury [37]. KOR agonist can decrease the proliferation of myeloma cells [38], while in other reports, KOR was linked to the increase of mesangial cell proliferation in kidney and angiogenesis [39,40]. Taken together, studies suggested that KOR can be linked to cell protection and proliferation, depending on the cell type. Interestingly, using this hypoxic neuronal cell model system, the increase of hKOR expression was a strong and long lasting adaptive event (lasting for 2 days at least) developing in surviving/attached neurons. Therefore, the functional role of hKOR in neuronal cells under the hypoxic condition is worthy of further examination. 

The other interesting point is that DFO differentially affects the opioid receptor expression. An increase of hKOR, a decrease of hMOR and no significant change of hDOR expression were simultaneously detected in surviving/attached NMB cells, implicating different functional roles of these opioid receptors upon DFO treatment. DOR has been reported to provide neuroprotection under hypoxia in an animal model [41], and the inhibition of TNF-α mediated inflammatory responses was also reported [42]. MOR is well-known to mediate analgesia and drug addiction, while its functional role under the hypoxic condition is controversial [43]. KOR, like the other opioid receptors, has the ability to change ion conductance leading to a decrease of neuronal excitability and calcium influx with the subsequent inhibition of neurotransmitter release [44,45]. KOR stimulation is also known to activate protein kinases, such as G-Protein Kinase (GRK) and MAPK (ERK1/2, JNK, PKC, p38 MAPK) pathway activation [46,47,48,49]. However, the pathway which KOR activates is highly dependent on the cell type, stimulus, and cell environment [48,49,50,51]. Therefore, it will be useful to further investigate the functional role of hKOR under the hypoxic condition in neuronal cells. 

## 4. Materials and Methods

### 4.1. Cell Culture

Human neuroblastoma NMB cells [20], grown in Roswell Park Memorial Institute (RPMI) medium (Invitrogen, Carlsbad, CA, USA) supplemented with 10% fetal calf serum (Atlanta Biologicals, Flowery Branch, GA, USA), were incubated at 37 °C with 5% CO_2_. 

### 4.2. Nuclear Extract Preparation

Nuclear extracts were prepared using NMB cells treated with or without DFO [52]. Cells grown to confluence were harvested, and washed with ice-cold phosphate-buffered saline. Cells were lysed using sucrose buffer, containing 0.32 M sucrose, 0.1 mM EDTA, 10 mM Tris-HCl, pH 8.0, 3 mM CaCl_2_, 2 mM magnesium acetate, 0.5% Nonidet P-40, 1 mM dithiothreitol, and 0.5 mM phenylmethylsulfonyl fluoride. Cell lysates were then microcentrifuged at 500× *g* at 4 °C for 5 min. The nuclei pellet was washed with sucrose buffer containing no Nonidet P-40, and then resuspended in the low salt buffer, containing 20 mM HEPES pH 7.9, 0.02 M KCl, 1.5 mM MgCl_2_, 25% glycerol, 0.2 mM EDTA, 0.5 mM phenylmethylsulfonyl fluoride and 0.5 mM dithiothreitol. The high salt buffer was then added to the nuclei. After 20 min incubation, 2.5 volumes of diluent was added, containing 25 mM HEPES, pH 7.6, 0.1 mM EDTA, 0.5 mM dithiothreitol, 0.5 mM phenylmethylsulfonyl fluoride, and 25% glycerol. Nuclear extract was obtained by microcentrifugation at 13,690× *g*. Aliquots of nuclear extract were stored at −80 °C.

### 4.3. SDS-PAGE and Western Blot Analysis 

Samples, separated by a 10% SDS-PAGE, were then transferred to the polyvinylidene difluoride membrane (GE healthcare, Pittsburgh, PA, USA). The membrane was incubated with the blocking solution, containing milk, and then further washed using 0.1% and 0.3% TTBS, containing Tween-20 either 0.1% or 0.3% in Tris-buffered saline, sequentially. The anti-HIF-1α antibody (Novus Biologicals, Littleton, CO, USA) was purchased. The enhanced ECL detection system (GE healthcare) was used. Signals were detected using a Storm phosphoimager (Molecular Dynamics, Caesarea, Israel). 

### 4.4. Generations of Various Constructs 

The fragments, containing individual or combination of HIF response elements of the *hKOR* gene, were synthesized and cloned into the pGL3-promoter vector (Promega, Madison, WI, USA). Mutagenesis was performed using synthesized fragments with the desired mutation, which were then cloned into the pGL3-promoter vector. The resulting plasmids were transformed into JM109 competent cells (Promega), respectively. Plasmids from each colony were isolated (Qiagene, Hilden, Germany) and then subjected to DNA sequencing to select the plasmid containing the correct sequences. 

### 4.5. RNA Isolation and RT-PCR 

Cells were lysed, and total RNAs were extracted using Tri-Reagent (Molecular Research Center, Cincinnati, OH, USA). First strand cDNA was synthesized using the reverse transcriptase (Invitrogen) with random primers at 37 °C for 50 min. PCR was carried out using 95 °C for 1 min, 68 °C for 35 s, and 72 °C for 35 s with a specific pair of primers. Primer sets for hMOR, hDOR, hHIF-1α and β-actin were described previously [6]. The primer pairs for hKOR are 5′-CCGATACACAAAGATGAAGACAGCAACC3′; 5′-GACATCGACGTCTTCCCTGACTTTGG-3′. The PCR products were examined using 2% agarose gel. The intensities of bands were quantified by Image Quant software. 

### 4.6. SiRNA Transfection 

Cells were first seeded in the 6-well plates. Cells were then transfected with the scrambled siRNA (Invitrogen) or HIF-1α siRNA (Ambien/ThermoFisher, Waltham, MA, USA) using lipofectamine RNAimax (Invitrogen). Following the manufacture’s protocol. Forty-eight hours after transfection, cells were harvested. 

### 4.7. Transient Transfection and Luciferase Reporter Assay 

Cells were transiently transfected with the pGL3-promoter plasmid containing a luciferase reporter and SV40 promoter or various plasmids as described in the text by lipofectamine (Invitrogen), based on the manufacturer’s protocol. The pCH110 plasmid, containing the β-galactosidase gene, was used as the internal control. After 48 h, transfected cells were harvested. Cells were thenlysed using the reporter lysis buffer (Promega). The reporter activity from each cell lysates was measured as relative light units (RLU) by Luminometer (Perkin Elmer, Waltham, MA, USA) and a Luciferase Assay system (Promega). 

### 4.8. Cell Membrane Preparation

Cells were harvested, resuspended, and homogenized using a buffer, containing 25 mM HEPES, pH 7.4, 1 mM EDTA, 0.32 M sucrose, 10 mM leupeptin, 0.1 mM phenylmethylsulfonyl fluoride, and 0.01% bacitracin). The cell membrane pellet was obtained by centrifugation at 1000× *g* and then 100,000× *g*, sequentially. The pellet was then resuspended in 25 mM HEPES, pH 7.4.

### 4.9. Competitive Opioid Binding Assay

Cell membranes (400 µg) were incubated with the radioisotope ligand, 2 nM [^3^H]-diprenorphine (Perkin Elmer), in 25 mM HEPES, pH 7.4, along with or without 1 µM unlabeled kappa agonist, U50, 488H (Sigma, St. Louis, MO, USA), for 10 min at RT. One µM of DADLE [d-Ala2, d-Leu5]-enkephalin and DAMGO (Sigma) were also included in every reaction in order to block the delta and mu opioid receptor bindings. The binding was stopped by the filtration method using GF/B filters. The filters were washed with 7% PEG in HEPES buffer, incubated with Scintiverse-BD (Fisher Scientific, Waltham, MA, USA) for 1 h at RT, and then subjected to counting using a beta-counter (Beckman Coulter, Brea, CA, USA). Data was normalized using the amount of proteins in control and treated cells.

### 4.10. Statistical Analysis

Values are reported as mean ± S.E.M. All experiments were repeated six times or more. Statistical significance was determined using the Student *t*-test.

## 5. Conclusions

We identified a new cellular adaptive response under hypoxia, a profound increase of hKOR expression, using the desferrioxamine (DFO)-induced hypoxia mimic model with human neuronal NMB cells. We have also elucidated its underlying mechanism. Two HIF response elements of the *hKOR* gene, located approximately at 1 Kb and 550 bp upstream of the start codon, were the keys to mediate the DFO-induced hypoxic effect. Mutation of both HIF elements completely abrogated the DFO-induced effect. Topological arrangement of both elements, likely in a head to tail or tail to head manner, can enhance the promoter activity synergistically. The functional relationship between HIF1 and hKOR expression was further confirmed using the HIF1α siRNA knock-down method. This study reported the adaptation of neuronal hKOR under hypoxia and revealed a newly identified mechanism of hKOR regulation, which provides additional insight hKOR under hypoxia for neuron protection and cancer progression.

## Supplementary Materials

Supplementary materials can be found at www.mdpi.com/1422-0067/18/1/211/s1.
