# Supplementary material for: Mechanism Governing Human Kappa-Opioid Receptor Expression under Desferrioxamine-Induced Hypoxic Mimic Condition in Neuronal NMB Cells
Source: Int J Mol Sci. 2017 Jan 20;18(1):211. doi: 10.3390/ijms18010211 (PMC5297840; doi:10.3390/ijms18010211)
Supplement: Supplementary file 1 [file ijms-18-00211-s001.pdf]

Supplementary Materials: Mechanism Governing Human Kappa-Opioid Receptor Expression under Desferrioxamine-Induced Hypoxic Mimic Condition in Neuronal NMB Cells

Jennifer Babcock, Alberto Herrera, George Coricor, Christopher Karch, Alexander H. Liu, Aida Rivera-Gines and Jane L. Ko

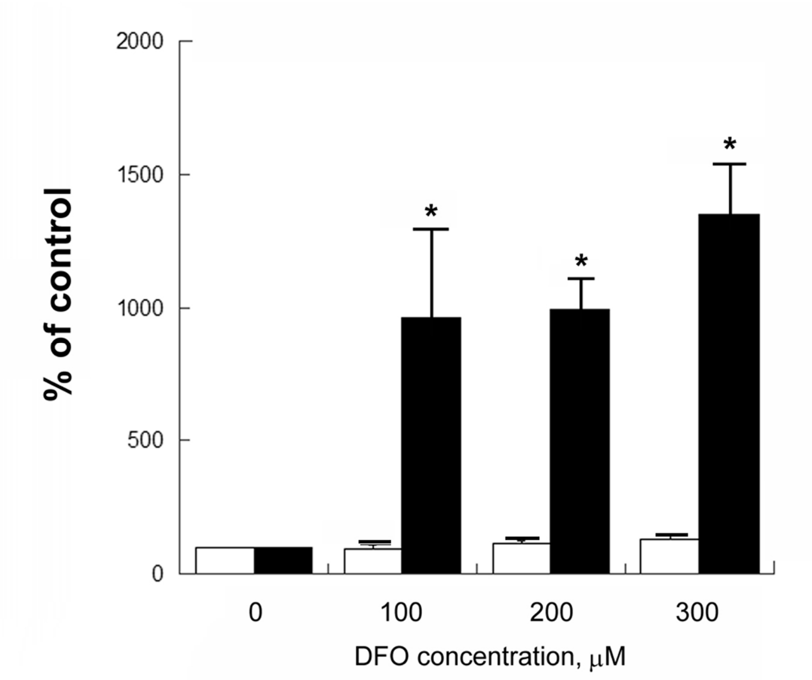

Figure S1. Effects of various DFO concentrations on *hKOR* gene expression using the luciferase reporter assay. NMB cells were transfected with the CD11 plasmid (black bars) and the pGL3-promoter vector (white bars), respectively. The pCH110 plasmid with the β-galactosidase was co-transfected and used as an internal standard. Transfected cells were treated without (0) or with DFO (100, 200 or 300 μM) for 24 h. Cells were harvested and subjected to luciferase assay. The promoter activity was expressed as a percentage of non-DFO treated control (0) activity, arbitrarily defined as 100%. Histograms represent mean values of activation. Error bars indicate S.E.M. Experiments were repeated six times. "*"indicates $p < 0.01$ (student *t*-test).

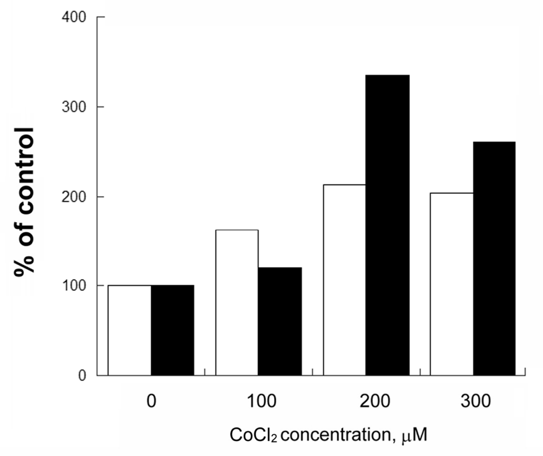

Figure S2. Effects of various $CoCl_2$ concentrations on *hKOR* gene expression using the luciferase reporter assay. NMB cells were transfected with the CD11 plasmid. The pCH110 plasmid with the β-galactosidase was co-transfected and used as an internal standard. Transfected cells were treated without (0) or with $CoCl_2$ (100, 200 or 300 μM) for 24 h. Cells were harvested and subjected to luciferase assay. The promoter activity was expressed as a percentage of non-treated control (0) activity, arbitrarily defined as 100%. Histograms represent the value of activation from individual samples (total of two samples: black bars and white bars).

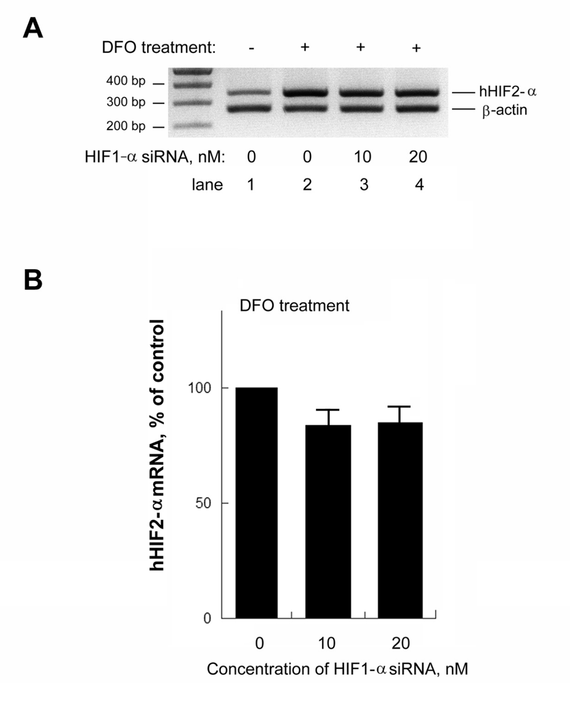

Figure S3. Examine the effect of hHIF-1α siRNA on the levels of hHIF-2α mRNAs. (**A**) NMB cells were transfected with hHIF-1α siRNA (lane 3, 10 nM; lane 4, 20 nM) or without hHIF-1α siRNA (indicated as 0 nM; lanes 1 and 2). Twenty-four hours after transfection, cells were treated without (lane 4, −) or with DFO for 24 h (lanes 1–3, +). RNAs were extracted from transfected cells. Semi-quantitative RT-PCR was carried out using a pair of human hHIF-2α primers. The β-actin primers were also included in every PCR reaction for normalization use. PCR products were separated by gel electrophoresis. DNA markers are labeled on the left; (**B**) quantitation of human hHIF-2α mRNA levels is shown, with the normalized hHIF-2α mRNA level from the DFO treated sample without siRNA as 100%. The hHIF-1α siRNA did not affect the amount of endogenous hHIF-2α mRNA. Histograms represent mean ± S.E.M. Experiments were repeated five times.
